# Growing demand for Bilateral Total Knee Arthroplasty: How to balance patient outcome with healthcare system challenges

**DOI:** 10.12669/pjms.40.9.10790

**Published:** 2024-10

**Authors:** Shaukat Ali Jawaid

Demand for Bilateral Knee Replacement Surgery wherein both knees are replaced during one surgical procedure is increasing the world over since it offers many advantages. However, careful patient selection is extremely important to ensure good outcome. It is also dependent on experience of the surgical team, the number of such procedures which are performed at that particular healthcare facility, the overall healthcare system and of course cost.

Total knee arthroplasty has evolved over the last five decades. It gives patients much better quality of life restoring their knee joint axes. There have been changes in the designs of knee implants and developments in instrumentation in addition to changes in preoperative and postoperative protocols which have all improved the surgical outcome to a great extent.^1^

Experiments with total knee arthroplasty started in 1970s. Beginning of total knee arthroplasty is considered an important development in the history of orthopedic surgery. A large number of orthopedic surgeons and biomedical engineers were involved in this process which has now developed into a multibillion industry with millions of knees implanted every year.

Historically Tgeophilus Gluck is credited with the first knee replacement surgery in the 1890s but these implants failed due to high infection and poor metallurgy besides inadequate fixation. Later on Dr. Waldius developed a hinge made of acrylic and in 1958 is reported to have manufactured it from cobalt chrome. This prosthesis remained in use until early 1970s, followed by Dr. Shiers (UK) and Guepar (France) prostheses.^2^,^3^

In Pakistan knee replacement arthroplasty is being successfully performed at many centers in major cities like Karachi, Lahore, Rawalpindi, Islamabad and Peshawar. However, BTKA is not very commonly performed at many centers due to various reasons. Syed Imran Bukhari & colleagues in their multi-center study which was based on data collected from Pakistan National Joint Registry from 2014-2021 have reported that 2,893 males and 6,577 females had total keen replacement surgery wherein Sindh province contributed 72% followed by Punjab 22.6% and KPK 4.7%. The reported co-morbidities were diabetes in 25.2% of patients while 69.2% suffered from hypertension. Majority of the implants used included Zimmer 29.5% followed by Biomet 12.5%, Johnson & Johnson 2.52% and Smith & Nephew 3.6%. They concluded that major factors which resulted in significant increase in total knee replacement were prevalence of arthritis, growing demand for greater mobility besides quality of life and effectiveness of joint replacement surgeries in the recent years.^4^

Advantages of Bilateral Total Knee Replacement Surgery include reduction in overall hospitalization and recovery period. Recovery period which includes prolonged physiotherapy sessions for couple of months can be more convenient for many patients and it also results in minimum disruption in normal routine. Moreover, combining the procedures in just one surgical sitting also reduces the overall cost tremendously related to hospital stay, anesthesia as well as fee of the surgical team. Patients can also save on out of pocket expenses due to single surgery.^5^,^6^ Other benefits include, early return to work and resuming daily activities.^5^,^6^

It has also been observed that bilateral knee replacement surgery ensures balanced rehabilitation ensuring symmetrical rehabilitation. Both knees heal and rehabilitate at the same time which avoids difference between the rehabilitation of one knee over the other. In case of single knee replacement surgery, the pressure is mounted on the other and when that is replaced, the outcome is sometime not the same as during this period lot of pressure is put on the other knee. There’s also just single exposure to potential risks associated with anesthesia, and surgery.^5^,^6^

However, despite all the above benefits bilateral knee replacement surgery is not suitable for every patient. One has to consider numerous factors like age of the patient, the overall health, fitness level and above all the presence of other comorbidities since majority of the elderly patients going for knee replacement surgery are suffering from multiple co-morbidities requiring polypharmacy. All the above factors play a vital role in determining which patient is suitable for this approach. Orthopedic surgeons undertaking these procedures rely on risk assessment scores and anesthesiologist input keeping in view the specific conditions and needs.

## Risks associated with bilateral Knee replacement surgery

This approach is not without risks. Along with benefits, there are certain risks and potential complications similar to any other major surgical procedure which must be kept in mind while taking any decision. There are increased surgical and anesthesia related risks. Surgical procedure takes much longer time, there is increased time the patient remains under anesthesia and all this can enhance the risks of complications related to anesthesia. Greater blood loss during surgery may necessitate increased blood transfusion which has its own risks. During the procedure there is greater stress on the heart and lungs which can be very risky for patients who are already suffering from cardiovascular disease. There are also increased risks of pulmonary complications which include pneumonia, pulmonary embolism because of prolonged immobility and extended surgical time. That is why early mobilization of the patient after the surgical procedure is advocated. Patients undergoing surgery at well-equipped healthcare facilities having experienced orthopedic surgeons ensure that the patient is helped to stand up the very next day and starts taking few steps with the help of walker within two to three days. During post-operative period there are risks of infection and the procedure of bilateral knee replacement may increase the risk.^7^ Hence, adequate infection control prior to surgery, good appropriate antimicrobials for few days to prevent infection are preferred. However, in high risk cases prolonged use of antibiotics may be considered. There is also risk of developing deep vein thrombosis or pulmonary embolism due to prolonged surgery time and immobility during the post-operative period.^8^,^9^

Intensive rehabilitation after this procedure is very challenging as both knees need to heal and strengthen simultaneously. To strengthen the muscles, intensive physical therapy is extremely important. Sometimes, patient ignore this important aspect which plays a vital role in overall outcome after the surgical procedure. In patients undergoing bilateral knee replacement surgery, pain management could be very difficult and complex. These patients are likely to experience more difficulty with mobility and daily activities during the initial phase of recovery hence they need much more assistance and support of the caring medical team and family members. In addition, undergoing prolonged surgery can be demanding psychologically. Recovery process can at times lead to feelings of depression or anxiety. This is particularly so in patients wherein the recovery is slow and more painful. Those patients already suffering from hypertension, diabetes with higher BMI might face higher risks. Elderly patients with lower ASA scores for surgery are likely to experience more complications and more challenges during the recovery period. It is extremely important that patients must have a thorough discussion with their orthopedic surgeons. The surgical team will evaluate their overall health, look at the medical history and finally decide if such an approach is appropriate and then develop a comprehensive surgical plan to minimize the potential risks.^10^

Today with advances in surgical techniques, anesthesia and pain management, the recovery in knee replacement surgery is much faster as compared to a decade ago. Healthcare facilities with high volume of joint replacement surgery should be selected since they have the facilities, expertise to manage the patients prior to surgery, during surgery and post operatively. Some facilities offer rehabilitation services as well which are part of the overall package while in other cases, the patients might have to acquire physiotherapy rehabilitation services on their own. After knee replacement surgery, most of the patients get rid of pain, get lot of relief and have tremendous improvement in their mobility.^11^

Majority of the patients recover within three to six months but may witness minor aches and pain from time to time. The patients who already have weakened muscles to begin with due to their limited activity before surgery may take longer to recover. According to meta-analysis approximately 82% of TKRs last 25 years.^12^ If patients lose weight before surgery and those who smoke give up smoking, it helps in faster recovery.^11^,^13^

Patients undergoing BTKA should be advised to start using the knee early, start standing up and walking with an assisted device like walker, use their new knee as early as possible. After return to their home, take longer walk gradually, start climbing stairs again gradually. Improve the range of their motion with exercise, step-ups on stairs, leg balances using single leg stance, do partial bending of their knee, raise their toe and heel while standing. However, they should be advised to avoid high impact activities during the first couple of months.^13^

Implants for knee replacement surgery come in various designs and materials and each has specific characteristics which are tailored to the patient needs. Right age for knee replacement surgery vary to a great extent on the individual’s condition, life style and overall health. Factors which influence timing for surgery include severity of knee pain and dysfunction, age of the patient. Middle age (defined differently across countries) is the predominant age bracket, wherein patients seek BTKR. However, there is no strict right age, decision is often based on severity of symptoms, overall health, and impact on quality of life.^12^
